# Appointment of Delegates to the National Convention

**Published:** 1846-03-01

**Authors:** 


*Appointment of Delegates to the National Convention.**—We learn that the State Society at the late session assumed the responsibility of appointing delegates to attend the convention, designating two in each senatorial district, and limiting their selection to the list of permanent members of the Society. We are sorry to find fault so soon with any thing pertaining to a movement to which we attach so much importance, and concerning which we have taken occasion frequently to express the hope that every thing would be harmonious and satisfactory. But we must say we received this information with surprise and regret. We understand the prime object of the convention to be to assemble representatives of the different views and interests of the profession in every section of our country. How could the state society know whether the permanent members designated for the several districts, actually represent the opinions and wishes of the majority of the medical men in their districts? What assurance had the society, that those appointed would attend the convention? What provisions are there in case the delegates appointed are unwilling or unable to attend, to supply substitutes? Why was it not left for the several medical societies to select their own delegates—those whom they prefer to send, and whom they would be able to ascertain would be willing to go? We make these inquiries without knowing what delegates have been designated except in our own district; and it is but justice to ourselves as well as to others, to disclaim any personal objections to the latter. It is the principle, not individuals that we oppose. Granting that it has so happened, that in all cases the selections are the very best that could have been made, we still have reason to complain of the course pursued by the state society. It ought to have referred the matter to the county societies, and it evinced a want of decent courtesy, if it be not an insult to the latter, that it did not. Were the profession throughout the state, to elect their representatives, they would feel that the action of the delegation from this state expressed the views of the majority of the profession in the state; but, as it is, they have no such assurance. It is easy to entertain the suspicion that members have been selected whose predilections are known to the fewwbo, it is said, manage the affairs of the state society; and we apprehend that this suspicion will be freely expressed as well as entertained. We hope that county societies will not fail, as it is, to elect delegates. We believe that they are fully competent to do this independently of the

* A meeting of the Society in this County is about to be called for this purpose.

state society. As regards this point, the convention can decide whether to discriminate or receive all. Our only object in this suggestion, is that if possible, no just grounds may exist for non-conformity with whatever action the convention may take on the subjects which will come before it.

